# Survival and success of zirconia compared with titanium implants: a systematic review and meta-analysis

**DOI:** 10.1007/s00784-023-05242-5

**Published:** 2023-09-23

**Authors:** Ninad Milind Padhye, Elena Calciolari, Anina Nives Zuercher, Sara Tagliaferri, Nikos Donos

**Affiliations:** 1 Centre for Oral Clinical Research, Institute of Dentistry, Faculty of Medicine and Dentistry, Queen Mary University of London (QMUL), London, UK; 2 Department of Medicine and Surgery, Centre of Dentistry, University of Parma, Parma, Italy; 3 Clinic of Reconstructive Dentistry, Centre of Dental Medicine, Zurich, Switzerland; 4 Department of Medicine and Surgery, University of Parma, Parma, Italy; 5 Center of Excellence for Toxicological Research, CERT, University of Parma, Parma, Italy

**Keywords:** Biological complications, Ceramic implant, Implant survival, Implant success, Marginal bone loss, Zirconium dioxide

## Abstract

**Objective:**

This systematic review assessed the available evidence on the survival and success rate of zirconia and titanium implants. As secondary outcomes, aesthetic, radiographic and clinical parameters, as well as biological and mechanical complications, were considered.

**Materials and methods:**

A systematic search was performed up to March 2022 to identify CCTs/RCTs comparing zirconia and titanium implants with a minimum of 12 months of follow-up. Meta-analysis was performed when ≥ 2 articles with similar characteristics were retrieved.

**Results:**

Four published articles with two RCTs (2 different patient populations) with 100 zirconia and 99 titanium implants that were followed up over 12–80 months were selected out of the 6040 articles. A non-statistically significant difference between zirconia and titanium implant survival at 12 months was suggested (*P* = 0.0938). The success rates were 57.5–93.3% and 57.1–100% for zirconia and titanium implants, respectively. The pink aesthetic score (PES) was higher for zirconia (10.33 ± 2.06 to 11.38 ± 0.92) compared to titanium implants (8.14 ± 3.58 to 11.56 ± 1.0).

**Conclusion:**

Based on the 2 RCTs retrieved in the literature, similar survival rates were reported for zirconia and titanium implants in the short term (12 months of follow-up). Future RCTs are warranted to evaluate the long-term outcomes of zirconia implants.

**Clinical relevance:**

Zirconia implants may be the procedure of choice, particularly in the aesthetic zone, since they show a similar survival and success rate as titanium implants on a short-term follow-up.

**Trial registration:**

Systematic review registration number**—**CRD42021288704 (PROSPERO).

**Supplementary Information:**

The online version contains supplementary material available at 10.1007/s00784-023-05242-5.

## Introduction

The replacement of missing teeth with dental implants is a widely accepted treatment procedure, with well-documented long-term stable results [1–3]. Introduced almost 40 years ago, commercially pure titanium is still considered the gold standard for intraosseous dental implants. Most implants are made from grade 4 titanium, while titanium alloys (Ti-6Al-4 V) are made from grade 5 titanium, which has greater fatigue resistance and strength [4]. Although titanium shows excellent mechanical properties and biocompatibility as an implant material, its disadvantages include potential discoloration of peri-implant soft tissue, risk of hypersensitive reaction and poor resistance towards peri-implantitis development [5, 6]. Moreover, a corrosion process has been reported when titanium was placed in contact with fluoride or metal alloys in the saliva [7]. It has also been suggested that bacterial biofilms could induce oxidation on the surface of titanium implants in an acidic environment, which in turn can elicit an inflammatory response [8, 9].

As a consequence of the increasing aesthetic standards in the field of implant dentistry and the concern of sensitivity to titanium, there has been a growing interest towards metal-free implant rehabilitations. Moreover, about 9.7% of implants display an aesthetic complication over a 5-year period [10]. These aesthetic complications can rise exponentially due to inappropriate implant position and angle of placement [11] or to a less-than-ideal keratinized mucosa architecture [12]. Ceramic material for manufacturing dental implants was introduced in the late 1960s [13]. The first generation of ceramic implants was made of alumina (aluminium oxide), due to its capability to osseointegrate with the native bone [14]. However, further research demonstrated the low fracture resistance and poor clinical performance of this material [15].

Zirconia (zirconium dioxide) was introduced in the 1990s into the field of dentistry, and it showed superior biomechanical properties compared to other ceramics [16]. Due to the phenomenon of allotropy, resulting in a phase transformation toughening mechanism, zirconia presents improved toughness and fracture resistance [17]. Following their introduction in the early 2000s, zirconia implants attracted significant interest, particularly for rehabilitations in the aesthetic zone [18]. Apart from their superior aesthetic properties, zirconia implants also display similar biocompatibility to titanium implants, lower affinity to plaque and low modulus of elasticity [19, 20]. The biomechanical properties of zirconia implants have been assessed in numerous experiments, and overall, their early failure rates seem generally higher compared with titanium implants. The early implant loss is mainly due to the lack of osseointegration [21]. However, robust data on long-term outcomes are missing [22].

Commercial zirconia implants were initially available only as one-piece implants. However, they displayed various limitations such as the lack of angled abutments to correct misalignment and the fact that cementation was the only option for connecting prosthodontic elements to one-piece implants, with the risk of leaving excess luting cement in the submucosal area, particularly when inserting the implants deeper in the aesthetic zone [15]. This led to the introduction of two-piece zirconia implants, where the fixtures and abutments are separated [23].

Previous systematic reviews have concluded that one- and two-piece zirconia and titanium implants demonstrate no significant difference in terms of bone-to-implant contact, removal torque or implant survival [24, 25]. Conversely, another systematic review by Elnayef in 2017 [26] reported that both one- and two-piece zirconia implants exhibited a lower survival rate and a higher marginal bone loss than titanium implants. However, this review did not specifically focus on comparative studies between zirconia and titanium implants and analysed solely the success and survival rates of zirconia implants. Hence, the present review aimed to systematically review the most recent evidence on the survival and success of zirconia as compared to titanium implants in order to provide relevant information for clinicians and set indications for their use in clinical practice by taking also into account the risk of biological and technical complications and aesthetic, clinical and radiographic outcomes.

## Materials and method

This review was conducted according to the Preferred Reporting Items for Systematic Reviews and Meta-Analyses (PRISMA) statement [27], and the protocol was compliant with the Cochrane Handbook [28]. The study protocol was registered in PROSPERO (registration number CRD42021288704).

### Focused question

The focus question for this review based on the PICOS [29] was, *Is there a difference in the survival and success rate of zirconia implants as compared with the titanium implants post 12 months of loading, as reported by randomised controlled studies and controlled clinical trials?*

### Inclusion criteria based on the PICOS

Inclusion criteria based on the PICOS were as follows:

- Type of participants (study population): systemically healthy, partially or completely edentulous subjects receiving one or more dental implants;
- Intervention (test group): zirconia implants;
- Comparison (control group): titanium implants;
- Outcomes: primary outcomes: implant survival and success (as defined by the authors of each study); secondary outcomes: marginal bone loss, implant failures, aesthetic outcomes (papilla fill index, pink aesthetic score (PES), visual analogue scale), patient-reported outcome measures (PROMs) and incidence of biological complications and adverse events, including peri-mucositis, peri-implantitis and implant mobility
- Types of studies: randomised controlled trials (RCTs) and case-controlled trials (CCTs) with a minimum of 10 patients per arm and followed up for ≥ 12 months post loading.

### Search method and database

A systematic search was performed in MEDLINE via OVID, Embase, Scopus and Cochrane Database (including the Central Register of Controlled Trials (CENTER)) in October 2021 and updated again on 31st March 2022 using the same search strategy developed by one reviewer and that combined MeSH term and free text (Appendix 1). The limitation to human studies was performed following the double negation strategy suggested by the Cochrane Handbook, i.e. combining the results with NOT (exp animals/ not humans.sh.). The Cochrane Highly Sensitive Search Strategy for identifying randomised trials was also applied. Bibliographies of review articles on this topic and of all studied included for data extraction were screened, and the database Scopus was used to identify all the papers that cited the included papers.

In the attempt to include both published and unpublished data, a specific thesis database, https://about.proquest.com/en/dissertations/ was searched. A hand search was performed for the last 2 years for the journals that published more about this topic and with a high impact factor (*Clinical Oral Implants Research*, *Clinical Implant Dentistry and Related Research*, *International Journal of Periodontics and Restorative Dentistry*, *Journal of Periodontology*, *Journal of Clinical Periodontology* were also performed). Grey literature was searched in http://www.greynet.org/opensiglerepository.html, and soon-to-be-published manuscripts were searched by contacting research groups with an interest in adjunctive therapies. Clinicaltrials.gov was investigated to identify potential ongoing or already completed RCTs/CCTs meeting the inclusion and exclusion criteria. Conference abstracts were excluded, and no language restrictions were applied.

### Study selection

A two-stage screening was carried out in duplicate and independently by two reviewers (NP, AZ). Studies were assessed based on their titles and abstracts first, and those studies that met the inclusion criteria were then screened for full-text analysis by the same independent reviewers. Any disagreement was resolved by discussion, and if necessary, a third reviewer (EC) was consulted. Reasons for study exclusion at the full-text stage were reported, and agreement at each of the two-stage screening processes was calculated using Kappa statistics.

### Data collection

Two reviewers (NP, AZ) independently extracted and recorded study data on ad hoc forms. In case of missing or unclear information, the authors were contacted by email to provide clarification or missing information. In case of missing or incomplete data and the absence of further clarification by study authors, data were excluded from the analyses.

In particular, the data extrapolated from each publication included authors’ names, year of publication, study design, trial registration, country of recruitment and treatment, funding status, population characteristics (age, gender, smoking status, dropouts), implant characteristics (implant system, dimensions, distribution in oral cavity, timing of implant placement, surgical approach) and prosthetic parameters (type of prosthetic rehabilitation, loading protocol, presence of provisional prosthesis). Moreover, data on the primary outcomes (implant survival and success and implant failure rate) and secondary outcomes (peri-implant clinical parameters including plaque score and bleeding on the probing score; radiographic marginal bone loss; incidence of biological and prosthetic complications, aesthetic scores) were extrapolated. Articles dealing with the same study population but simply reporting on different follow-ups were grouped together during data extraction as indicated in the Cochrane Handbook [23].

### Risk of bias evaluation

Quality assessment and risk of bias for all included papers were conducted by two reviewers (NP, EC) following the recommendations of the *Cochrane Handbook for Systematic Reviews*. As such, the risk of bias in non-randomised studies of interventions (ROBINS-I) tool was employed for CCTs, and the revised Cochrane risk-of-bias tool for randomised trials (RoB 2) (updated October 2018) was employed for RCTs [30]. Recommended algorithms were followed to reach both domain-level and overall judgement of risk of bias of included studies. The funding bias was assessed by evaluating if the authors disclosed potential conflicts of interest and sources of funding for the study carried out. For articles dealing with the same study population but simply reporting on different follow-ups, one single risk of bias assessment was performed.

### Measures of treatment effect and unit of analysis

For the primary outcomes (implant survival, implant success), mean values along with 95% confidence intervals were used to summarise data for each treatment group. The unit of analysis was the implant. Quantitative data analysis was performed considering implant survival after 12 months of follow-up. Implant survival was obtained as the number of available implants at the follow-up divided by the number of implants placed at baseline. We calculated the survival standard deviation by extrapolating the square root of the variance, according to the formula p*q*n/*n* − 1, where *p* is the probability of success (surviving implant), *q* the probability of failure (*q* = 1 − *p*), and *n* represents the number of implants placed. We compared titanium and zirconia implant survival by calculating the differences between the means of the two implant groups (titanium vs. zirconia) and their 95% confidence interval (CI). Combined analyses were assessed through a random effect approach, and the forest plot was drawn with StatsDirect 3.2.7 (StatsDirect Ltd). Heterogeneity was assessed using the *I*-squared tests. Given the small number of studies included, the publication bias was not assessed [31], and StatsDirect did not allow to estimate the 95%CI of *I*-squared for the same reason. A *p* value < 0.05 was considered statistically significant.

## Results

**Fig. 1:**
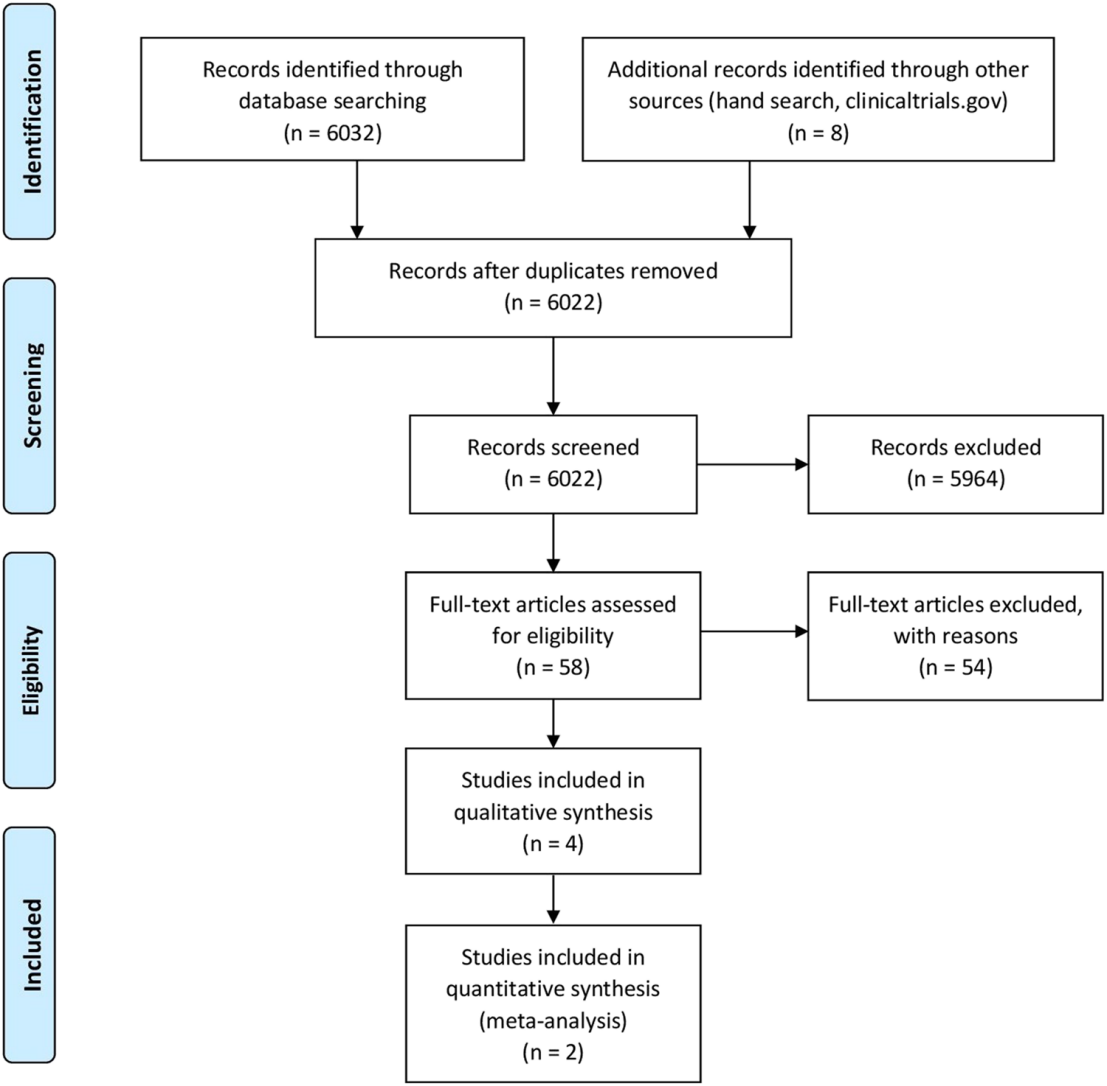
Study flow diagram

A total of 6040 potentially relevant articles was initially identified. After the first-stage screening based on titles and abstracts, 5982 articles were excluded, and 58 articles were selected for full-text analysis. After full-text assessment, 54 articles were excluded (reasons for exclusion can be found in Appendix 2), and 4 articles meeting the inclusion/exclusion criteria were included for the qualitative assessment (Fig. 1). Kappa of agreement was > 0.8 at both screening stages. Although 4 articles were included, they referred to the same 2 clinical trials with different follow-up periods.

The characteristics of the included studies can be found in Table 1. All 4 articles were RCTs published between 2014 and 2020, and they were carried out in a university setting in Austria and New Zealand. One trial [28, 29] was funded by the implant company producing the tested implants, while the other trial [26, 27] reported no fundings. Overall, the included studies reported on a total of 46 patients with 100 zirconia and 99 titanium implants. One hundred three implants were placed in the maxilla, while 96 implants were placed in the mandible. The follow-up period ranged from 12 to 80 months. All the implants were placed in a healed extraction socket (type IV placement) with a 2-stage approach and loaded conventionally. While two articles (one trial) used one-piece zirconia and titanium implants, where the patients were rehabilitated using implant-supported overdentures [32, 33], the remaining two articles (one study) used two-piece zirconia and titanium implants, where the participants were rehabilitated using a single implant-supported zirconia crown [34, 35]. While one trial [28, 29] excluded smokers, no information on the smoking status was available on the other trial [26, 27].

**Table 1:** Demographic characteristics of the included studies

Author and year	Study design	Trial registration	Country study conducted	Funding	No. subjects (dropout)	Age (range)	Gender (M/F)	Smokers included (yes/no)	Type of rehabilitation	Loading protocol
Osman et al. (2014) [32], Siddiqi et al. (2015) [33]	RCT, parallel arm	NS	New Zealand	No	24 (5 dropouts)	46–80	15/4	NS	Implant-supported complete overdenture	Conventional
Payer et al. (2015) [35], Koller et al. (2020) [34]	RCT, parallel arm	NS	Austria	Study supported by Ziterion GmbH, Uffenheim, Germany	22	24–77	13/9	No	Implant-supported single crown	Conventional

Author and year	No. implants		Implant dimension		Provisional prosthesis	Timing of implant placement	One-/two- piece implant	Distribution		
	Zirconia	Titanium	Length (mm)	Diameter (mm)				Zirconia	Titanium	
Osman et al. (2014) [32], Siddiqi et al. (2015) [33]	84 (Southern implants (Irene, South Africa))	84 (Southern implants (Irene, South Africa))	6, 8, 10, 11.5	3.8	Yes, but not implant supported	Type IV	One-piece implant	Off-centre implant in incisal region: 12 Bilateral implants in premolar region: 24 Mid palatal implant: 12	Off-centre implant in incisal region: 12 Bilateral implants in premolar region: 24 Mid palatal implant – 12	
Payer et al. (2015) [35], Koller et al. (2020) [34]	16 (Ziterion ® vario z, Ziterion GmbH)	15 (Ziterion ® vario t, Ziterion GmbH)	10, 11.5, 13	4	No	Type IV	Two-piece implant	Max inc: 3 Max mol: 0 Mand prem: 1 Mand mol: 12	Max inc: 2 Max mol: 2 Mand prem: 1 Mand mol – 10	

### Primary outcomes

**Fig. 2:**
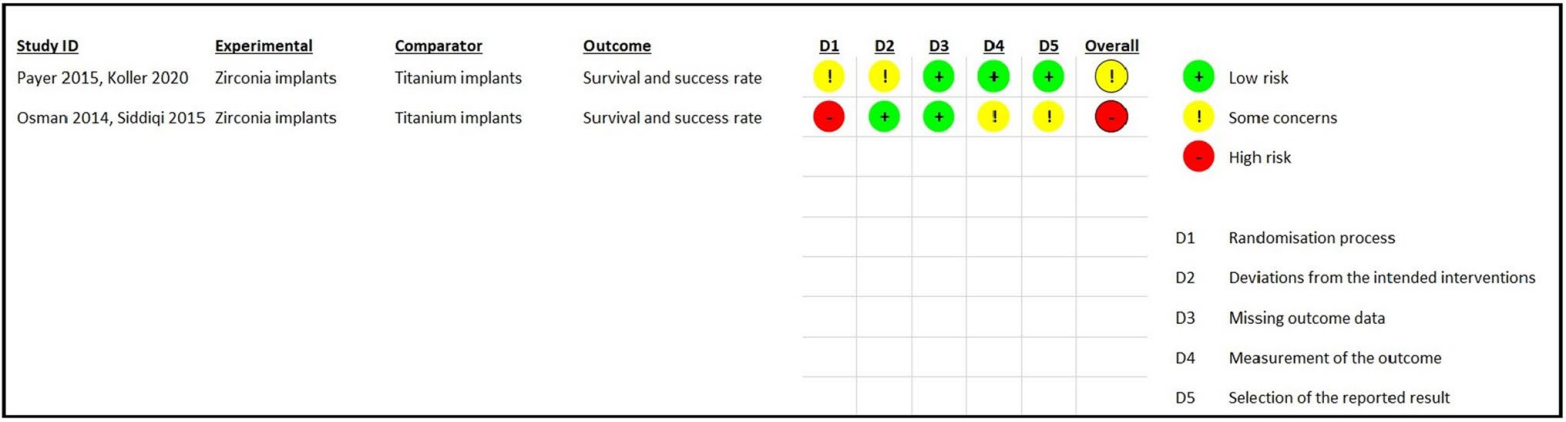
Risk of bias assessment according to Rob2

Survival rates ranged from 67.6 to 93.3% and 66.7 to 100% for zirconia and titanium implants, respectively (67.6 to 90.9% for one-piece zirconia implants and 66.7 to 95.8% for one-piece titanium implants; 85.7 to 93.3% for two-piece zirconia implants and 93.3 to 100% for two-piece titanium implants respectively) (Table 2). A higher number of early implant failures, within 1 year after loading, (15 out of 84) were noted for one-piece zirconia implants compared to titanium implants (2 out of 84). Despite a trend for lower survival of zirconia implants suggested by the included studies, meta-analysis obtained by combining the 12-month follow-up data provided by Osman et al. [26] and Payer et al. [35] showed a non-statistically significant difference between the two types of implants (*P* = 0.0938, Fig. 2).

**Table 2:** Primary outcomes of included studies

Author and year	Implant type	Follow-up period (months)	Survival rate (one-piece implants)		Success rate (one-piece implants)		Failures (*n*)				Marginal bone loss (mm) (mean ± SD)	
			Zirconia	Titanium	Zirconia	Titanium	Zirconia		Titanium		Zirconia	Titanium
							Early	Late	Early	Late		
Osman et al., (2014) [32]	One-piece zirconia and titanium implant	12	71.2%*	82.1%*	57.5%*	57.1%*	15	6	2	8	0.42 ± 0.4	0.18 ± 0.47
Siddiqi et al. (2015) [33]		12	67.6%*	66.7%*	67.6%*	66.7%*	12	9	2	8	NS	NS
Payer et al. (2015) [35], Koller et al. (2020) [34]	Two-piece zirconia and titanium implant	12	93.3%	100%	93.3%	100%	0	1	0	0	1.16 ± 1.01	0.88 ± 0.56
		18	93.3%	100%	93.3%	100%	0	1	0	0	1.2 ± 0.76	1.15 ± 0.73
		24	93.3%	100%	93.3%	100%	0	1	0	0	1.48 ± 1.05	1.43 ± 0.67
		30	85.7%	93.3%	85.7%	93.3%	0	2	0	0	1.51 ± 0.68	0.92 ± 0.72
		80	85.7%	93.3%	85.7%	93.3%	0	2	0	1	1.38 ± 0.81	1.17 ± 0.73

Although Osman et al.’s and Siddiqi et al.’s study reported on the same population and follow-up, they applied different methods for reporting on survival and success, hence they are here presented in two separate rows

Since the criteria applied to calculate implant success were heterogenous amongst the studies, no meta-analysis was performed. Nevertheless, the success rates tended to be lower for zirconia compared to titanium implants, ranging from 57.5 to 93.3% and from 57.1 to 100%, respectively (67.6 to 57.5% for one-piece zirconia implants and 66.7 to 57.1% for one-piece titanium implants; 85.7 to 93.3% for two-piece zirconia implants and 93.3 to 100% for two-piece titanium implants) (Table 2).

### Secondary outcomes

Secondary outcomes are detailed in Table 3. Marginal bone loss for zirconia implants ranged from 0.42 ± 0.4 mm to 1.51 ± 0.68 mm (0.42 ± 0.4 mm for one-piece zirconia implants and 1.16 ± 1.01 mm to 1.51 ± 0.68 mm for two-piece zirconia implants), while for titanium implants it ranged from 0.18 ± 0.47 mm to 1.43 ± 0.67 mm (0.18 ± 0.47 mm for one-piece titanium implants and 0.88 ± 0.56 mm to 1.43 ± 0.67 mm for two-piece titanium implants). Bleeding scores around zirconia implants ranged from 0.34 ± 0.42% to 16.43 ± 6.16%, while for titanium implants they ranged from 0.26 ± 0.36% to 15.46 ± 6.57%.

**Table 3:** Secondary outcomes of included studies

Author and year	Implant type	Follow-up	Plaque index % (mean ± SD)		Bleeding on probing % (mean ± SD)		Peri-implantitis (*n*)		Fracture (*n*)		Pink aesthetic score (mean ± SD)	
			Zirconia	Titanium	Zirconia	Titanium	Zirconia	Titanium	Zirconia	Titanium	Zirconia	Titanium
Osman et al. (2014) [34], Siddiqi et al. (2015) [35]	One-piece zirconia and titanium implants	12 months	0.44 ± 0.49	0.46 ± 0.47	0.34 ± 0.42	0.26 ± 0.36	NS	NS	3	0	NS	NS
Payer et al. (2015) [35], Koller et al. (2020) [34]	Two-piece zirconia and titanium implants	12 months	15.88 ± 6.67	11.19 ± 5.69	11.9 ± 9.44	7.9 ± 4.98	0	0	0	0	10.33 ± 2.06	9 ± 3.54
		18 months	11.9 ± 4.77	14.13 ± 4.77	7.6 ± 6.15	14.3 ± 3.89	0	0	0	0	11 ± 2	8.14 ± 3.58
		24 months	19.38 ± 0.88	16.05 ± 8.29	9.1 ± 4.34	7.4 ± 3.39	0	0	0	0	11.22 ± 1.56	10.75 ± 0.71
		30 months	23.68 ± 10.74	21.04 ± 6.09	10.05 ± 6.43	15.46 ± 6.57	0	0	0	0	11.38 ± 0.92	11.14 ± 1.07
		80 months	11.07 ± 8.11	15.2 ± 15.58	16.43 ± 6.16	12.6 ± 7.66	0	1	0	0	11.11 ± 1.27	11.56 ± 1.01

From the available data, only 1 titanium implant showed signs of peri-implantitis at the 80-month follow-up period, while 3 one-piece zirconia implants had mechanical complications (implant fracture at 1-year follow-up). The PES ranged from 10.33 ± 2.06 to 11.38 ± 0.92 and 8.14 ± 3.58 to 11.56 ± 1.01 for two-piece zirconia and titanium implants, respectively.

Although our protocol included other secondary outcomes, such as interdental papilla fill, visual analogue scale and PROMs, no data were available in this respect from the included clinical trials.

### Risk of bias

**Fig. 3:**
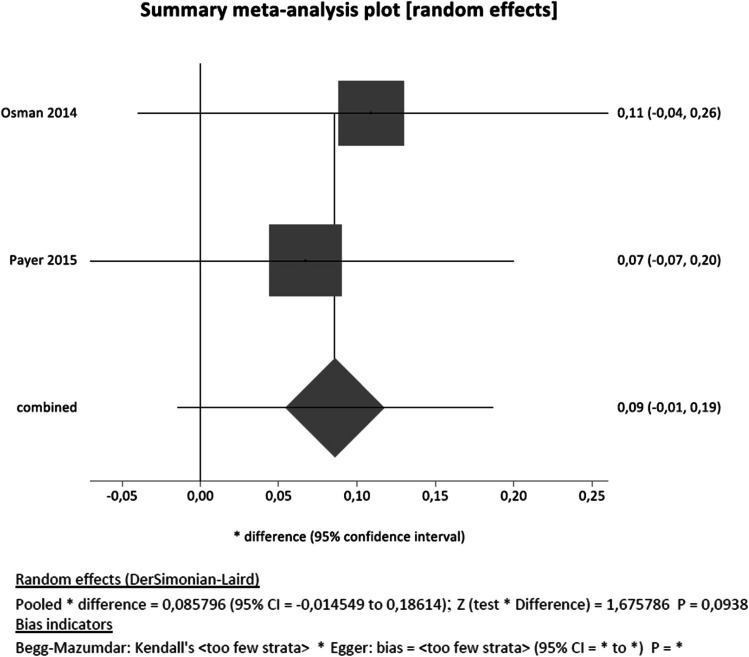
Forest plot performed to assess the weighted mean difference in implant survival between titanium and zirconia implants at 12 months of follow-up. The random effect model was applied

Risk of bias assessment was done using the ROB-2 assessment tool (Fig. 3). Overall, one trial showed a high risk [26, 27], while the other [28, 29] raised some concerns. In particular, there was a likelihood of bias due to an inadequate description of the randomisation and allocation process in both studies [26–29]. Since the operators and assessors could not be blinded, a possible bias could also arise from the measurement of outcome (1 out of 2 studies). Remarkably, one study [29] received support (financial support and/or materials) from the implant manufacturers.

## Discussion

This systematic review aimed to compare the survival and success rates of zirconia and titanium implants with a minimum of 12 months of follow-up after functional prosthetic loading. In contrast to previous systematic reviews that focused on animal studies or non-comparative studies [16, 36, 37], this review was the first to compare zirconia and titanium implants based only on CCTs and RCTs. Four articles (RCTs) were identified in the literature matching the inclusion criteria. However, 2 articles followed up on the same sample population, and therefore, we ended up including 4 articles reporting data only from 2 exclusive patient populations. Despite the obvious limitations of doing a meta-analysis with only 2 studies, the combination of the only 2 RCTs available in the literature with 12-month follow-up data can offer clinicians some valuable information on what is the level of evidence available (poor) in the literature at the moment. In particular, the quantitative synthesis indicated a non-statistically significant difference in implant survival for zirconia compared to titanium implants (Fig. 2). This outcome needs to be interpreted with caution, owing to the limited number of studies available, the combination of different implant systems (one-piece vs. two-piece) supporting different types of rehabilitations (overdentures vs. single-crowns) and of different implant locations. In particular, it is important to highlight the unconventional implant distribution reported in two studies [32, 33] where implants were employed to support overdentures, as the behaviour of such implants may not be comparable to implants placed to support single cemented crown [38]. Our review and meta-analysis also clearly indicated important knowledge gaps in the field of the use of zirconia implants and the need for long-term RCTs to understand the performance and clinical outcomes associated with such implants.

Previous literature [2] suggested that titanium implants may present with a relatively higher survival and success rate than zirconia implants over a short-term follow-up, but we lack long-term controlled studies that could clarify the superiority of one implant material over the other. Nevertheless, in the included studies, the survival rate for zirconia implants was lower than the 5-year survival rate for titanium implants with single crown (97.2%), as reported in previous systematic reviews [39, 40]. This may be attributed to the higher biological complications around zirconia as compared to titanium implants, particularly a higher rate of marginal bone loss [22]. Kohal et al. [41] demonstrated that one-piece zirconia implants demonstrated > 2 mm bone loss in 40% of the patients and > 3 mm bone loss in 28% of the patients at the 1-year follow-up post-prosthetic loading. On the other hand, one-piece titanium implants displayed 0.9 mm marginal bone loss after 1 year [42].

One-piece implants have a rough body for intraosseous placement, while a machined smooth collar emerges through the soft tissues. On the other hand, two-piece implants are designed for submerged healing, which claims to reduce the initial bone resorption [43]. Also, it has been noted that detachment and repositioning of the abutments result in around 0.2 mm marginal bone resorption [44]. Amongst zirconia implants, one-piece systems seem to have a lower survival and success rate than two-piece systems. It is important to note that in the study [26, 27] that assessed the one-piece implants placed in completely edentulous arches to support an overdenture, 3 implants were placed on the ridge, while 1 implant was placed in the mid-palatine region for all the patients. The majority of early implant failures in such study, particularly for zirconia implants, occurred in the mid-palatine implants (42.1%) [26]. It can be speculated that the unconventional implant site might have been the reason for the high number of early failures of one-piece implants, due to the movements of the tongue.

In a randomised trial by Cannizzaro et al. [45], a survival rate of 87.5% was noted for two-piece zirconia implants with immediate loading at 12 months of follow-up. However, the different implant placement and loading protocols (immediate placement, immediate loading) might have caused a higher rate of failure in these implants. Similarly, a survival rate of 87% was reported by Cionca et al. [46], wherein most of the two-piece implant failures occurred during the early healing period. Our systematic review also reported a similar survival rate for zirconia implants (85–94%), with the exception of the study by Siddiqi et al. [33], which included mid-palatine implants and reported a survival rate of 67.6%.

While implant survival was well defined, implant success criteria were heterogeneously described in the included studies. The studies by Payer et al. [35] and Koller et al. [34] considered success criteria as no peri-implant translucency, no implant-related pain, infection or paraesthesia, no implant fracture and intact support of prosthetic restoration, while Osman et al. [26] and Siddiqi et al. [33] considered lack of mobility, pain and neuropathy as the criteria for success. Although a meta-analysis could not be performed because of the aforementioned heterogeneity of criteria applied, a tendency for lower implant success in zirconia implants was suggested, particularly when looking at the data of two-piece implants (85.7 to 93.3% success for two-piece zirconia implants vs. 93.3 to 100% for two-piece titanium implants).

While previous short-term studies indicated success rates for zirconia implants ranging from 93 to 100% [47, 48], our review showed more heterogeneous data, with zirconia success rates ranging from 57.5 to 93.3% over 12–80 months of follow-up following loading. This is likely due to the longer follow-up of the included studies, the heterogeneity in the position of the implant placements and the potential effect of combining the outcomes of one-piece and two-piece implant data. As previously mentioned, the combination of crestal and mid-palatine implants [26, 27] might have skewed the results of our systematic review; however, separate data based on implant positioning were not available.

Over the past 4 decades, titanium has often been considered the material of choice for dental implants due to its high survival rate (97.2% at 5 years and 95.2% at 10 years), coupled with biocompatibility, low corrosion and high strength [49, 50]. However, a 5-year cumulative aesthetic complication rate of 7% has been noted amongst titanium implants [48]. An increased aesthetic demand, particularly in the anterior region, is the reason why zirconia implants are now being considered as a valuable alternative treatment, particularly in subjects with a thin gingival phenotype. With an aesthetic complication rate close to 0%, zirconia implants are the obvious choice for anterior aesthetic rehabilitations [45]. As a matter of fact, two studies (same population) [28, 29] in this review showed a tendency for higher PES for zirconia compared to titanium implants. A high PES (≥ 8) was also noted in maxillary single-tooth anterior zirconia implants in a study by Kniha et al. [51]. From the patients’ perspectives, papilla fill for one-piece zirconia implants was also found to be quite satisfying in an analysis by Hollander et al. [52]. Since the remaining two studies (same population) [26] included in this review used an implant overdenture, the PES was not recorded; hence, it is not possible to infer a superiority of zirconia implants in terms of aesthetic scores based on the included RCTs.

Zirconia implants have shown to present a lower inflammation rate compared to titanium implants due to lower bacterial attachment [53]. In our review, only one case of peri-implantitis was reported for the titanium group and no cases for the zirconia group (Table 3). The bleeding indices showed ambiguous results between the two groups, without a clear advantage for the zirconia group (Table 3). Owing to heterogeneity in the methods of assessing the outcomes, a statistical comparison was not performed, as such no meaningful conclusions could be drawn in this respect.

This systematic review presents some limitations. Since research for zirconia implants is in its incipient stage and still considered an emerging treatment modality, most available literature on this topic is based on animal model studies, case series or in vitro analyses. Considering that RCTs and CCTs offer the best evidence for therapy efficacy and effectiveness [54], we only included such trials in our review. However, to date, only short-term (12 to 80 months) controlled studies are available to compare zirconia and titanium implants, thus preventing to draw any robust conclusion. Moreover, although the studies included for this systematic review were RCTs, they did present a few shortcomings in their study design, including a lack of detailed inclusion and exclusion criteria, accountability of patient dropouts and information on soft tissue architecture. Furthermore, the studies used implants with different thread designs and surface characteristics, as well as prosthetic superstructures, thus making them challenging to compare. More importantly, the clinical outcome assessment for implants placed in the midpalatal region for overdenture retention (Osman et al. [32], Siddiqi et al. [33]) may not be comparable with implants placed in the edentulous ridge, therefore the outcomes obtained by combining such implants risk being misleading. Finally, it is important to note that while one brand of zirconia implants employed in one trial (Ziterion) is still available in the market, to the best of our knowledge, the other implant brand (Southern) is no longer marketed. Due to this shortcoming, while the aim of our review was to compare the outcomes associated to the different implant materials per se (without considering the implant brand), the generalisation of our findings can be limited. Due to the continued evolution of the implant market and associated biomaterials, it is important that future studies will focus on currently available zirconia implants, in order to provide more clinically relevant and applicable information.

Future investigations are warranted to compare the long-term survival and success outcomes, along with any potential late mechanical and biological complications. It would be relevant also to assess the effects of guided bone regeneration (and regenerative procedures in general) in association with zirconia implants. It is also suggested that three-dimensional radiographic assessments should be considered in future studies to allow a 3D evaluation of peri-implant bone stability, including buccal and lingual/palatal bone levels around zirconia implants. Moreover, it is also recommended that patient-reported outcome measures are investigated [32], since patients’ request for superior aesthetic outcomes may be one of the main reasons for opting for zirconia over titanium implants.

## Conclusion

There is a limited number of CCTs/RCTs in the literature comparing titanium versus zirconia implants,; hence, no robust conclusions can be made. Despite a trend for increased failure rate in zirconia implants, results from the current review do not support an increased survival rate of titanium compared to zirconia implants in the short term (12 months). While zirconia implants may potentially be advantageous in the aesthetic area, no clear advantage could be inferred based on the few RCTs retrieved in the literature. Likewise, the limited data available do not allow us to draw robust conclusions in relation to the prevalence of biological complications and inflammatory scores. A higher incidence of fracture for zirconia implants was reported in one study. Future RCTs controlling for confounding factors and considering clinical, radiographic and patient-reported outcomes are needed to evaluate the long-term success and performance of zirconia implants.

### Supplementary Information

Below is the link to the electronic supplementary material.

Supplementary file1 (DOCX 16 KB)

Supplementary file2 (DOCX 17 KB)

## Data Availability

Additional data are available in the Supplementary File.
