# Supplementary material for: Survival and success of zirconia compared with titanium implants: a systematic review and meta-analysis
Source: Clin Oral Investig. 2023 Sep 23;27(11):6279–90. doi: 10.1007/s00784-023-05242-5 (PMC10630218; doi:10.1007/s00784-023-05242-5)
Supplement: Supplementary file 1 — Supplementary file1 (DOCX 16 KB) [file 784_2023_5242_MOESM1_ESM.docx]

1. Search strategy for OVID

|  | MeSH terms | Free text |
| --- | --- | --- |
| Population | Exp Dental Implants/  OR exp Dental Implantation OR Dental Prosthesis, Implant‐Supported/ OR jaw, edentulous, partially OR osseointegration | ((osseointegrated adj2 implant$) and (dental or oral))  OR dental implant$  OR (implant$ adj5 dent$)  OR ((crown$ or bridge$ or prosthes$s or restoration$) and implant$) OR "implant supported dental prosthesis"  OR ("blade implant$" and (dental or oral))  OR ((endosseous adj2 implant$) and (dental or oral))  OR ((dental or oral) adj2 implant$) OR (implant-supported and prosthes$s) |
| Intervention/Comparison | Zirconium OR exp surface properties OR exp ceramics | Zirconium dioxide OR (zirconia and implant*) OR (implant and surface) OR (ceramic and implant) OR (ZLA and surface) or (metal-free and implant) |

This subject search was linked to the Cochrane Highly Sensitive Search Strategy (CHSSS) for identifying randomised trials in MEDLINE: sensitivity‐ maximising version (2008 revision) as referenced in Chapter 6.4.11.1 and detailed in box 6.4.c of *The Cochrane Handbook for Systematic Reviews of Interventions,* Version 5.1.0 (updated March 2011).

1. Randomized controlled trial.pt.

2. controlled clinical trial.pt.

3. randomized.ab.

4. placebo.ab.

5. drug therapy.fs.

6. randomly.ab.

7. trial.ab.

8. groups.ab.

9. or/1‐8

10. exp animals/ not humans.sh.

11. 9 not 10

2. Cochrane library via CENTER

|  | MeSH terms | Free text |
| --- | --- | --- |
| Population | Exp Dental Implants/  OR exp Dental Implantation OR Dental Prosthesis, Implant‐Supported/ OR jaw, edentulous, partially OR osseointegration | ((osseointegrated adj2 implant$) and (dental or oral))  OR dental implant$  OR (implant$ adj5 dent$)  OR ((crown$ or bridge$ or prosthes$s or restoration$) and implant$) OR "implant supported dental prosthesis"  OR ("blade implant$" and (dental or oral))  OR ((endosseous near/2 implant$) and (dental or oral))  OR ((dental or oral) near/2 implant$) OR (implant-supported and prosthes$s) |
| Intervention/Comparison | Zirconium OR exp surface properties OR exp ceramics | Zirconium dioxide OR (zirconia and implant*) OR (implant and surface) OR (ceramic and implant) OR (ZLA and surface) or (metal-free and implant) |

3. Search strategy for EMBASE

|  | MeSH terms | Free text |
| --- | --- | --- |
| Population | Exp tooth implant OR exp Tooth Implantation OR exp osseointegration OR exp edentulousness OR exp dental prosthesis and implant | ((osseointegrated adj2 implant$) and (dental or oral))  OR dental implant$  OR (implant$ adj5 dent$)  OR ((crown$ or bridge$ or prosthes$s or restoration$) and implant$) OR "implant supported dental prosthesis"  OR ("blade implant$" and (dental or oral))  OR ((endosseous near/2 implant$) and (dental or oral))  OR ((dental or oral) near/2 implant$) OR (implant-supported and prosthes$s) |
| Intervention/Comparison | Exp zirconium dioxide OR exp surface property OR exp dental ceramics | Zirconium dioxide OR (zirconia and implant*) OR (implant and surface) OR (ceramic and implant) OR (ZLA and surface) or (metal-free and implant) |

This subject search was linked to the RCT strategy based on SIGN filter3, amended to embase.com format:

'clinical trial'/de OR 'randomized controlled trial'/de OR 'randomization'/de OR 'single blind procedure'/de OR 'double blind procedure'/de OR 'crossover procedure'/de OR 'placebo'/de OR 'prospective study'/de OR 'randomi?ed controlled' NEXT/1 trial* OR rct OR 'randomly allocated' OR 'allocated randomly' OR 'random allocation' OR allocated NEAR/2 random OR single NEXT/1 blind* OR double NEXT/1 blind* OR (treble OR triple) NEAR/1 blind* OR placebo*

4. Search strategy for SCOPUS

| Population | ((osseointegrated adj2 implant$) and (dental or oral))  OR dental implant$  OR (implant$ adj5 dent$)  OR ((crown$ or bridge$ or prosthes$s or restoration$) and implant$) OR "implant supported dental prosthesis"  OR ("blade implant$" and (dental or oral))  OR ((endosseous near/2 implant$) and (dental or oral))  OR ((dental or oral) near/2 implant$) OR (implant-supported and prosthes$s) |
| --- | --- |
| Intervention | Zirconium dioxide OR (zirconia and implant*) OR (implant and surface) OR (ceramic and implant) OR (ZLA and surface) or (metal-free and implant) |
