# Supplementary material for: Survival and success of zirconia compared with titanium implants: a systematic review and meta-analysis
Source: Clin Oral Investig. 2023 Sep 23;27(11):6279–90. doi: 10.1007/s00784-023-05242-5 (PMC10630218; doi:10.1007/s00784-023-05242-5)
Supplement: Supplementary file 2 — Supplementary file2 (DOCX 17 KB) [file 784_2023_5242_MOESM2_ESM.docx]

Reasons for study exclusion

| Authors | Year | Reason for exclusion |
| --- | --- | --- |
| Al-Nawas et al | 2012 | Titanium-zirconium (Roxolid) implants analyzed |
| Al-Zordk et al | 2020 | Finite element analysis |
| Bambini et al | 2013 | Immunohistochemical analysis |
| Benic et al | 2013 | Titanium-zirconium (Roxolid) implants analyzed |
| Bienz et al | 2021 | Follow-up <12 months |
| Borg et al | 2014 | Titanium and zirconia framework compared |
| Bradley et al | 2021 | No comparison with titanium implant |
| Cabrera-Domínguez et al | 2020 | No comparison with titanium implant |
| Chandra et al |  | Study still in patient recruitment phase |
| Chen et al |  | Study still in patient recruitment phase |
| Cionca et al | 2017 | No patient data |
| Cruz et al | 2020 | In vitro analysis |
| da Rocha et al | 2021 | In vitro analysis |
| de Sanctis et al | 2009 | Animal model study |
| Depprich et al | 2014 | No patient data |
| Franchi et al | 2007 | Animal model study |
| Hadi et al | 2018 | In vitro analysis |
| Herrmann et al | 2016 | Retrospective analysis |
| Ioannidis et al | 2015 | Titanium-zirconium (Roxolid) implants analyzed |
| Kniha et al | 2020 | Retrospective analysis |
| Kniha et al | 2021 | No patient data |
| Kniha et al | 2021 | Not in English |
| Koch et al | 2010 | Animal model study |
| Kohal et al | 2009 | In vitro analysis |
| Kohal et al | 2009 | Animal model study |
| Kohal et al | 2013 | In vitro analysis |
| Kollar et al | 2008 | Case series |
| Kubasiewicz-Ross et al | 2018 | Animal model study |
| Kuo et al | 2017 | Animal model study |
| Leite et al | 2020 | In vitro analysis |
| Lopez et al | 2018 | In vitro analysis |
| Martins et al | 2018 | Animal model study |
| Rodriguez et al | 2017 | In vitro analysis |
| Mobilio et al | 2013 | In vitro analysis |
| Muller et al | 2015 | Titanium-zirconium (Roxolid) implants analyzed |
| Moller et al | 2012 | Animal model study |
| Oliva et al | 2007 | No comparison with titanium implant |
| Oliva et al | 2008 | No comparison with titanium implant |
| Osman et al | 2012 | No clinical parameters assessed |
| Quirynen et al | 2015 | Titanium-zirconium (Roxolid) implants analyzed |
| Rimondini et al | 2002 | In vitro analysis |
| Roehling et al | 2019 | Animal model study |
| Rosentritt et al | 2014 | Zirconia versus titanium abutment comparison |
| Rottmar et al | 2019 | In vitro analysis |
| Scarano et al | 2004 | Follow-up <12 months |
| Schwarz et al | 2015 | Animal model study |
| Siddiqi et al | 2016 | Animal model study |
| Siddiqi et al | 2016 | No clinical parameters assessed |
| Sollazzo et al | 2008 | No comparison with titanium implant |
| Stadlinger et al | 2010 | Animal model study |
| Tete et al | 2009 | Animal model study |
| Thoma et al | 2015 | Animal model study |
| Thoma et al | 2016 | Animal model study |
| Tolentino et al | 2013 | Titanium-zirconium (Roxolid) implants analyzed |
| Zhou et al | 2007 | No comparison with titanium implant |
| Zipprich et al | 2019 | Animal model study |
